# Building Research Infrastructure to Develop Greater Learning Efficiencies (BRIDGE)

**DOI:** 10.3233/SHTI231141

**Published:** 2024-01-25

**Authors:** Danne C ELBERS, Nathanael R FILLMORE, Jennifer LA, Hannah M TOSI, Samuel AJJARAPU, Rupali DHOND, Karen MURRAY, Danielle VALLEY, Colleen SHANNON, Mary T BROPHY, Nhan V DO

**Affiliations:** a VA Boston Healthcare System, Boston MA, USA; b Harvard Medical School, Boston MA, USA; c Boston University School of Medicine, Boston MA, USA

**Keywords:** Learning health system, oncology

## Abstract

In this manuscript, we outline our developed version of a Learning Health System (LHS) in oncology implemented at the Department of Veterans Affairs (VA). Transferring healthcare into an LHS framework has been one of the spearpoints of VA’s Central Office and given the general lack of evidence generated through randomized control clinical trials to guide medical decisions in oncology, this domain is one of the most suitable for this change. We describe our technical solution, which includes a large real-world data repository, a data science and algorithm development framework, and the mechanism by which results are brought back to the clinic and to the patient. Additionally, we propose the need for a bridging framework that requires collaboration between informatics specialists and medical professionals to integrate knowledge generation into the clinical workflow at the point of care.

## 1. Introduction

Multiple models for implementation of Learning Health Systems (LHS) have been proposed to meet the mandate issued by the Institute of Medicine (IOM) to transform healthcare systems. Inherent to all of them are three essential infrastructure activities supporting a learning cycle. These are (A) the creation of clinical knowledge bases to integrate and manage a growing volume of diverse data types, (B) the generation of actionable knowledge using real-world evidence and advanced analytics and (C) the delivery, application and iterative adaptation of these discovered insights (knowledge) to improve patient care. Each of these core activities encompasses multiple informatics approaches and technological challenges. In the learning cycle described by Friedman [1], there is often a disconnect between the research activities that generate knowledge and the clinical operational activities seeking to effectuate knowledge to improve care.

Unfortunately, it may take years for a research discovery to become standard of care [2,3]. This delay, called the “bench to bedside” gap, exists for both traditional clinical-trial-type research and newer data science approaches [4,5]. The LHS as proposed in 2007 by Etheredge [6] and the IOM [7] offers a framework to narrow this divide. Since then, the Department of Veterans Affairs (VA) made the development, integration, and adoption of an LHS one of their priorities [8]. Not all problems need to be solved through an LHS, but the oncology domain is highly suitable given the general lack of evidence generated through randomized control clinical trials to guide medical decisions. In 2016, the investigators at the Boston VA Cooperative Studies Program (CSP) published two articles that together presented a vision for the creation of an oncology LHS within the VA [9,10]. They emphasized a need to enable existing technologies, noting that the VA already houses a large integrated electronic health record (EHR) system and advanced data computing capabilities. Although many publications describe motivations, barriers, and opportunities for utilizing an LHS framework, examples implementing all three infrastructure components described above are limited [3,11]. Here we report our efforts known as BRIDGE (Building Research Infrastructure to Develop Greater Learning Efficiencies) to close the gap between research and clinical care.

## 2. Methods

### 2.1. Infrastructure to Manage Data

The creation of an actionable knowledge base is critical when instantiating an LHS to build algorithms or clinical guidelines. This requires investment in the development of data repositories and data sharing infrastructure. The integration of multi-modal patient data also requires longitudinal assurance of data quality and privacy. In 2016 we started the development of the Precision Oncology Data Repository (PODR) [12,13]. This repository includes EHR data, targeted tumor sequencing data, proteomic, metabolomic, and medical imaging data including computed tomography (CT) scans and digital pathology slides. Furthermore, to share PODR data for further research purposes, we established the Research Precision Oncology Program, which provides a mechanism for patients to consent to broad data sharing [13].

### 2.2. Infrastructure to Generate Actionable Knowledge

Galvanizing multi-modal data into actionable knowledge is the next critical step for any successful LHS. The scope can vary significantly depending on the domain and goals. Our activities to generate knowledge for precision oncology include, but are not limited to, automated cohort identification, hypothesis generation, and predictive algorithms for prognosis, adverse effects, and responses to therapy.

### 2.3. Infrastructure to Deliver and Apply Knowledge to Improve Patient Care

The delivery and application of actionable knowledge to improve outcomes is the final step in the LHS cycle. Not only does this require informatics tools that can mobilize new knowledge into readily consumable structures, but it also requires continuous monitoring and integration of treatment decisions and outcomes back into knowledge bases for future re-use. To achieve this, we have developed a platform that hosts a suite of applications facilitating workflows for trial matching and virtual tumor boards.

To explore opportunities for clinical decision support or use of predictive algorithms, our team utilizes a user-centered process and agile development with coordinators and clinicians. Users provide vital feedback, highlight opportunities for workflow automation, and identify information gaps amenable to knowledge delivery.

## 3. Results

**Figure 1.**
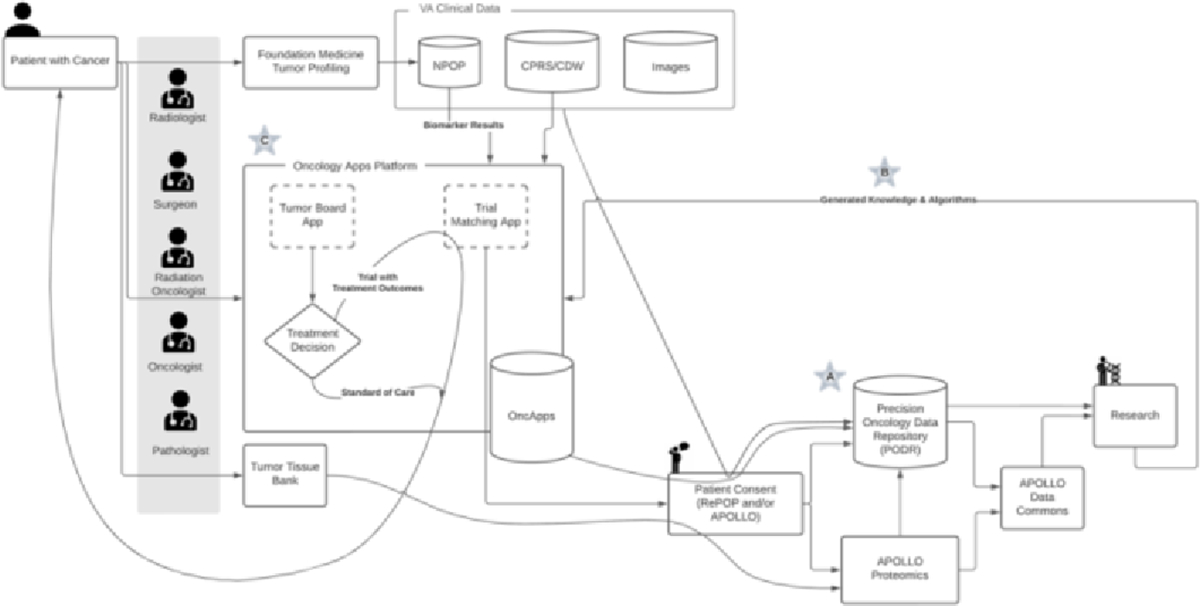
Precision Oncology Learning Healthcare System at the Department of Veterans Affairs.

Figure 1 illustrates our integration of the three key LHS technology infrastructures (A, B and C) to help bridge the “bench to bedside” gap. Data collected during routine care or clinical trials are transformed through research into knowledge for clinical decision making. When a VA patient is diagnosed with cancer, their multi-modal data is collected throughout the VA and, with consent, integrated into PODR. To date, de-identified data sets on 200,000+ veterans have been securely shared with external academic partners. Using this real-world evidence, our data scientists and collaborators have generated descriptive studies [14], observational studies [15], and risk predictions models [16]. When our clinicians identify a research study that potentially has clinical impact, we follow up with further development as a clinical decision support tool. As an example, EHR data and Natural Language Processing (NLP) techniques were used to extract lung cancer tumor descriptions from patient records and develop a “frailty index” to identify patients at risk for negative outcomes [17]. This frailty index is now being incorporated as an application in our Oncology Applications platform.

The LHS framework is ideal for a personalized approach to the patient instead of the population. For example, for tumor board workflows it incorporates a precision medicine approach by facilitating treatment recommendations based on molecular profile using the Molecular Oncology Almanac [18] and patient similarity networks (PSN) method.

Integrating observational studies or clinical trials within the process of routine clinical settings is another aspiration to bridge research and clinical care. We have laid the foundation to support such activities with our applications to manage workflow of pragmatic trials [19] and our trial matching application developed as part of the BRIDGE effort screening for eligible patients. Our trial matching application is in use for 19 oncology trials in the VA.

## 4. Discussion

An LHS approach can help bridge the gap between research and clinical care. However, implementation can be challenging, and application must be considered within the context of the broader institutional culture, available resources, and patient population. Our current efforts continue and have already involved multiple pilot projects over the past 6 years with our community of learners under the vision for VA oncology described by Fiore et al. [9,10]. Indeed, the full benefit of an LHS approach can only be achieved when new research advances become rapidly accessible at all points of patient care. Given the importance of technology to achieve this goal, there are examples (both in-house and commercial) of ongoing efforts for each of the three technology-centric LHS elements. Unlike these other efforts, the BRIDGE ecosystem not only integrates relevant technologies but also includes a research and clinical user base committed to continual improvement and expansion of its applications.

The limitation of our current effort is lack of real-time data access and integration with our EHR. However, a virtual tumor board workflow for delivering knowledge products can work well under these constraints, as 24 hours’ delay is adequate for tumor boards, and the workflow does not require seamless integration with our EHR. Our future work will involve methods for real-time data access. Evaluation of the impact of the research product within the clinical workflow may require further evaluation by a clinical trial or prospective studies. Although we have established basic infrastructure to quickly identify trial cases and support workflow for pragmatic trials, funding will be needed to further extend capabilities to pursue impact studies.

## 5. Conclusions

The delay between new medical discoveries and when those discoveries are put into practice at a patient’s bedside is unacceptable for our Veterans with cancers. We are hopeful that our BRIDGE effort described here will provide the technologic tools necessary to unite our research and clinical communities under an LHS framework to provide the best care possible.
